# Modification of MWCNTs with Bi_2_WO_6_ nanoparticles targeting IL-1β and NLRP3 inflammasome via augmented autophagy

**DOI:** 10.1371/journal.pone.0309389

**Published:** 2024-10-31

**Authors:** Zenaa R. Rahoomi, Duha S. Ahmed, Majid S. Jabir, Haney Samir, Ayman A. Swelum

**Affiliations:** 1 Ibn Sina University of Medical and Pharmaceutical Science, Baghdad, Iraq; 2 Department of Applied Sciences, University of Technology- Iraq, Baghdad, Iraq; 3 Department of Veterinary Medicine, Tokyo University of Agriculture and Technology, Fuchu, Japan; 4 Department of Animal Production, College of Food and Agriculture Science, King Saud University, Riyadh, Saudi Arabia; National Research Centre, EGYPT

## Abstract

This study reports the facile hydrothermal synthesis of pure Bi_2_WO_6_ and Bi_2_WO_6_\MWCNTs nanocomposite at specific molar ratio 1:2.5 of Bi_2_WO_6_:MWCNTs and elucidates their role in modulating the NLRP3 inflammasome pathway via autophagy induction. Comprehensive characterization techniques, including XRD, Raman, UV.Vis PL,FESEM,EDS and TEM, revealed the successful incorporation of MWCNTs into the Bi_2_WO_6_ structures, leading to enhanced crystattlinity, reduced band gap energy (2.4 eV) suppressed charge carrier recombination and mitigated nanoparticles aggregation. Notably, the reduced band gap facikitaed improved visible light harvesting, a crucial attribute for photocatalytic applications. Significantly, the nanocompsoite exhibited a remarkable capacity to augment autophagy in bone marrow-derived macrophages (BMDMs), consequently down-regulating the NLRP3 inflammasom activation and IL-1β secretion upon LPS and ATP stimulation. Immunofluorescence assays unveiled increased co-localization of LC3 and NLRP3, suggestion enhanced targeting of NLRP3 by autophagy. Inhibition of autophagy by 3-MA reversed these effects, confirming the pivotal role of autophagy induction. Furthermore, the nanocomposite attenuated caspase-1 activation and ASC oligomerzation, thereby impeding inflammasome assembly. Collectively, these findings underscore the potential of Bi_2_WO_6_\MWCNTs nanocompsite as a multifaceted therapeutic platform, levering its tailored optoelectronic properties and sbility to modulate the NLRP3 infalmmasome via autophagy augmentation. This work covers the way for the development of advanced nanomaterials with tunable functionalities for combating inflammatory disorders and antimicrobial applications.

## 1. Introduction

Among the pathways that are connected with described as a highly selective cellular clearance system, autophagy is responsible for the maintenance of cellular and tissue homeostasis [1–4]. The process of autophagy is very important in a variety of physiological activities, including cellular maintenance, response to stress, and regulation of energy metabolism. In addition, the autophagy process consists of four key steps: The initiation, the expansion of membranes and the formation of autophagosomes, the fusion of autophagosomes with lysosomes, and the destruction of products inside autophagic lysosomes are all characteristics of this process [5–8]. Moreover, Cellular autophagy is often considered a protective process used to protect both the inside and outside of the cellular environment from potential damage within organisms when the cell sustains injury from external causes. In addition, autophagosomes possess two distinct characteristics: double- mitochondria and fragments of the endoplasmic reticulum are examples of cytoplasmic components that have been incorporated into the layered membrane structure of the living cell. The elongation of the membrane inside autophagic vesicles results in the creation of distinctive autophagosomes, which are characterized by their double-membrane architectures [9, 10]. Various conditions contribute to the initiation of autophagy in organisms, including oxidative stress, and pathogen invasion. Instead of being spherical, the structure has more of a flattened shape, resembling a concave structure made up of lipid bilayers which is a significant criterion for identifying autophagy by electron microscopy. Furthermore, the mammalian target of rapamycin, (mTOR) is a crucial gene that controls autophagy. Nanoparticles have received more attention because of their high mechanical features as well as chemical stability, and biocompatibility [6, 7]. Besides, nanoparticles are being explored to modulate autophagy for therapeutic benefits owing to the large surface area, chemical stability, and the ability to pass through cell membranes. Carbon-based nanoparticles like fullerenes, nanotubes, graphene, and their derivatives are often used in biomedical settings, where they have been effectively exploited for medication and tissue regeneration. It has been shown that almost all carbon-based nanomaterials exhibit antibacterial properties. Additionally, carbon-based nanomaterials can regulate autophagy by causing damage to membranes and oxidative stress [11, 12]. In particular, multi-walled carbon nanotubes; and MWCNTs have demonstrated encouraging prospects in the removal of antibiotic residues in aqueous solutions due to their excellent adsorption performance, outstanding reusability, and good stability. Due to the reduction of ban gap of semiconductor in the hybrid, it will be promosing to absorb visible light, which will generate charge carriers (e-h) in semiconductor. The free radicals O^2-^ and OH formed can react with organic substance inside bacterial cell membrane, leading to death of the bacteria [13]. Among Aurivillius oxide materials, Bi_2_WO_6_ represent an excellent material as a semiconductor photocatalyst [8, 9]. Moreover, Bi_2_WO_6_ suffers from a relatively wide bandgap (e.g., 2.80 eV) and, consequently, exhibits low visible light absorption capability [12]. Bi_2_WO_6_ is considered a photocatalytic degradation of organic pollutants and antibacterial agents [14–16]. High photo-generated charge carriers result in an increasing recombination rate in Bi_2_WO_6_, which hinders its practical applications to a great extent and improves photocatalytic activity in pollution removal [17, 18]. Moreover, the enhancement of its activity can be achieved using different strategies such as surface sensitization, ion doping, and interaction with semiconductors of small band gaps such as organic dyes, metal clusters, and MWCNTs. The aim of this work is represented by studying the influence of addition of Multi-Walled Carbon Nanotubes (MWCNTs) to Bi_2_WO_6_ via the hydrothermal technique. Besides, the XRD spectrum showed that addition MWCNTs increased the crystallinty of the Bi_2_WO_6_\MWCNTs nanocomposite compared to pure Bi_2_WO_6_ with smaller crystallite size to provide higher surface to volume ratio as well as improved by FESEM and TEM. The optical properties by using UV.Vis, PL and Raman analysis of nanocomposite exhibited a red shifting in absorption edge, indicating better visible light absorption due to reduction band gap after adding MWCNTs and suggestion lower (e-h) recombination rates which favorable for photocatalyitc activity and forming O^-2^ and -OH radicles as ROS species. In the current study, we are prepared of MWCNTs and Bi2WO6 as nanocomposite. Furthermore, we used them as autophagy inducer in BMDMs. Bi2WO6/MWCNTs nanocomposite reduce the level of IL-1β, and NLRP3 inflammasome while increasing autophagy. The results suggested that Bi2WO6/MWCNTs enhance autophagy in two different possible mechanisms. The first one is targeted IL-1, caspase-1, and ASC activation, while the second is NLRP3 degradation. These findings raise the possibility that the Bi2WO6/MWCNTs nanocomposite could be an effective therapy for reducing inflammation through the increase of autophagy.

## 2. Materials and method

### 2.1 Materials

By utilizing high-quality chemical reagents and materials sourced from reliable suppliers. Bismuth (III) nitrate pentahydrate (Bi (NO_3_)_3_⋅5H_2_O) 98% pure was procured from the German company Sigma-Aldrich. Ammonium metavanadate (NH_4_VO_3_) is 99% supplied by the UK-based Glentham.com. The Chinese company Chemical Reagent Com. supplied the sodium hydroxide (NaOH). We bought Multi-Walled Carbon Nanotubes (MWCNTs) from the American company Grafton, which has a purity level of 90% and sizes between 8 and 15 nm. We purchased 69% pure nitric acid (HNO_3_) from the CDH Company in India. All experimental approaches relied only on deionized water as the emulsifier.

### 2.2 Synthesis of pure Bi_2_WO_6_ and Bi_2_WO_6_/ MWCNTs nanocomposite

The hydrothermal approach was used to manufacture pure Bi_2_WO_6_ [19, 20]. Initially, 80 ml of deionized water (D.W.) was mixed with Bi(NO_3_)_3_.5H_2_O (2 mmol), sodium fluoride Na_2_WO_4_.2H_2_O (1 mmol), and 0.05g of CTAB as catalyst agent. After 30 minutes of stirring at room temperature, this mixture was strained using a magnetic stirrer and adjusted to a pH of 7. Subsequently, by using a 100 ml autoclave lined with Teflon, the white suspension solution was sealed. Next, after sealed, the autoclave was maintained at 180°C in a convection oven for 16 hrs. Afterward, the autoclave was allowed to cool to room temperature, causing a white precipitate to be formed. Bi_2_WO_6_ was cleaned several times using ethanol and distilled water, which was then dried in a convection oven set at 60°C for 10 hours. For Bi_2_WO_6_\MWCNTs nanocomposite, the molar ratio of Bi_2_WO_6_: MWCNTs was set as 1:2.5. Initially, 1 gram of functionalized F-MWCNTs and 0.4 gram of Bi_2_WO_6_ were added to deionized water placed for 50 ml, this mixture was stirred for 1hr in a beaker using a magnetic stirrer. To achieve dispersion, the mixture was dissolved using ultrasonication for 2 hours. Simultaneously, 2 ml of 4 mol/L sodium hydroxide (NaOH) dissolved in deionized water after 20 minutes of magnetic stirring at 25°C. The Bi_2_WO_6_\MWCNTs until the pH reached 7, while the solution was constantly agitated; the (NaOH) solution was added dropwise at a rate of 3 drops per minute. After the mixture was formed, it was heated for 16 hr at 180°C in an autoclave made of stainless steel with a Teflon lining that could contain 150 ml. Lastly, the autoclave was given time to cool down to ambient temperature. We gathered the byproducts, rinsed them with ethanol and water many times, and then allowed them to dry for 12 hours at 60°C in a convection oven.

### 2.3 Defining characteristics

The crystalline composition of pure Bi_2_WO_6_ and Bi_2_WO_6_/MWCNTs nanocomposite products, respectively was examined using an XRD diffractometer (XRD 6000, Shimadzu), with CuK radiation (λ = 1.542Å), the data that was acquired fell between the range of 10° to 60°, with a 2θ value. To conduct photoluminescence (PL) spectroscopy, a monochromatic excitation wavelength of 250 nm was used at ambient conditions to examine the emission wavelength (Cary Eclipse fluorescence model, Iran) around the range of 200–800 nm. The features of the optical were performed using UV-visible spectroscopy (Shimadzu UV-1800 spectrophotometer). Raman spectra were measured at room temperature using (Takram N1-541). Filed emission scanning electron microscope images (FE-SEM, TESCAN, MIRA3) of pure Bi_2_WO_6_ and Bi_2_WO_6_/MWCNTs nanocomposite samples were captured to study their morphology. Besides, EDS stands for energy-dispersive X-ray spectroscopy was conducted during FESEM measurements as a relatively simple but powerful technique for the elemental analysis of samples. For more details, a transmission electron microscope (TEM, Philips-EM-208S) was performed to examine the morphology of materials.

### 2.4 Isolation of BMDMs

To separate primary bone marrow-derived macrophages (BMDMs), male C57/BL6 mice aged 8–10 weeks were used as described in [21, 22].

### 2.5 Immunofluorescence assay

BMDM cells were seeded onto two-well polycarbonate Lab-Tek slides. BMDMs were subjected to 500 μg/mL of LPS for 12 hours and 2.5 mM of ATP for 30 minutes, both in the presence and absence of prepared NPs. After twice washing the cells in 1X PBS, they were fixed for 30 minutes at room temperature using 2% PFA. Permeabilization with 0.5% Triton-X for 30 minutes at room temperature and blocking with 5% normal goat serum for 60 minutes are the following steps. After that, the cells were incubated for 24 hours at 4°C with 1 μg/mL of the primary rabbit polyclonal anti-LC3, rabbit polyclonal anti-NLRP3, and rabbit polyclonal anti-ASC antibodies. PBS was used twice to wash the BMDMs. After that, the cells were incubated for two hours at room temperature (RT) with 1 μg/mL of the secondary antibodies, goat anti-rabbit IgG or goat anti-mouse IgG conjugated with Alexa Fluor 568. PBS was used to wash the cells three times. Ultimately, a confocal microscope was used to analyze them [23].

### 2.6 Flow cytometry assay for LC3, NLRP3, and caspase-1

Antibodies and Fc receptors inhibited non-specific Fc-mediated binding for 30 minutes at 4°C using rat anti-mouse CD16/CD32 antibody at a concentration of 1 μg/mL. Flow cytometry was utilized to quantify LC3, NLRP3, and Caspase-1 (FACS Calibur flow cytometer, BD) [24].

### 2.7 Enzyme-linked immunosorbent assay

Following the manufacturer’s instructions, the cytokine concentration of mouse IL-1 (Cat. No. ab1977742; Abcam, Cambridge, MA, USA) was determined using an enzyme-linked immunosorbent assay (ELISA) reagent. At 570 nanometers, the absorbance was measured with an ELISA plate reader [25].

### 2.8 Statistical analysis

Statistical analysis was performed on the acquired data using an unpaired t-test in GraphPad Prism. The mean values with standard deviations were provided [26, 27].

## 3. Results and discussions

### 3.1 Defining characteristics

**Fig 1:**
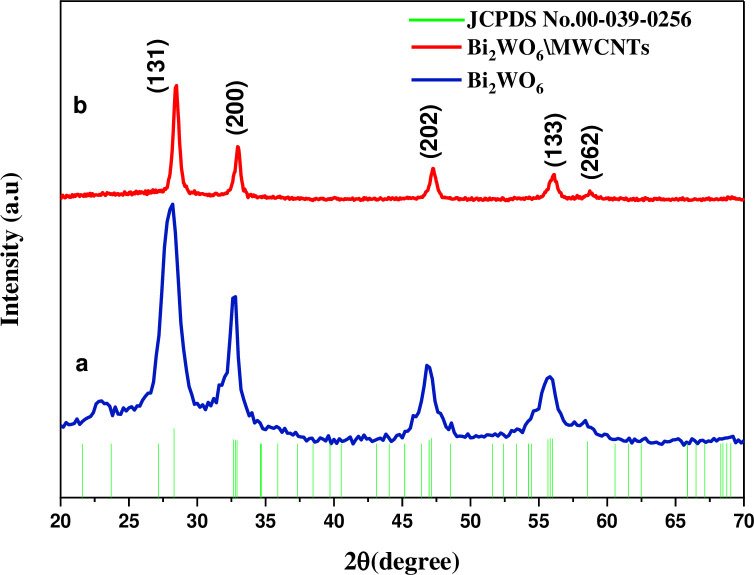
The patterns of XRD of a) Bi_2_WO_6_ pure and b) nanocomposite Bi_2_WO_6_/MWCNTs at specific molar ratio of Bi_2_WO_6_: MWCNTs 1:2.5. by using the hydrothermal method.

The XRD spectrum of pure Bi_2_WO_6_ and Bi_2_WO_6_/MMCNTs nanocomposite was carried out at ambient temperature, respectively as shown in Fig 1(A) and 1(B). Fig 1(A) illustrates the XRD patterns of Bi_2_WO_6_ through stronger diffraction peaks at 2θ values of 28.3°, 32.91°, 47.13°, 55.99°, and 58.54°. These peaks were related to the (131), (200), (202), (133), and (262) planes crystallographic, respectively, agreed with the reference code (JCPDS card No. 00-039-0256). In the state of MWCNTs, the standard diffraction peaks at 2θ = 26.0° and 2θ = 43.20° correspond to planes (002) and (100), respectively, and in good agreement with (JCDPS card No. 01–0646 [28]. On the other hand, the XRD pattern of Bi_2_WO_6_/MWCNTs nanocomposite at a specific molar ratio 1:2.5 of Bi_2_WO_6_:MWCNTs was characterized by its sharpness and narrower half-peak width as shown in Fig 1(B). These observations collectively provide evidence that the inclusion of MWCNTs enhances the degree of crystallinity shown by the nanocomposite material. These results demonstrate the successful embedding of MWCNTs within the Bi_2_WO_6_ structure. To determine the average crystallite size, which is designated as D = k λ/ (β cosθ), the Scherrer formula was used where k represents the crystal shape factor, λ is the wavelength of the Cu-K radiation that was utilized, and θ is the Bragg angle as shown in Table 1. It becomes clear that the intensity of the nanocomposite began to gradually decrease, whereas the intensity of pure Bi_2_WO_6_ was higher as shown in Fig 1(A) and 1(B). The reason for the decrease in the intensity of the direction because that the embedded MWCNTs worked to create their atomic levels within the crystal lattice of Bi_2_WO_6_ and nanocomposite. Accordingly, the atomic levels of both the atoms of pure and nanocomposite material will not work to scatter the X-rays falling on them in the same phase, and this causes interference during the synthesis reaction and the peak’s intensity diminishes after the embedded MWCNTs, this decrease in grain size causes an increase in the diffraction angle [29, 30]. Since the nanocomposite containing MWCNTs material can quench luminescence or other forms of intensity this could explain the observed decrease compared to pure Bi_2_WO_6_ as shown in the XRD analysis and as improved by the PL spectrum.

**Table 1:** Crystalline size of Bi_2_WO_6_ and nanocomposite at a specific ratio (1:2.5) Bi_2_WO_6_: MWCNTs nanocomposite by using hydrothermal method.

Materials	2Ө (deg)	hkl	FWHM (deg)	d-spacing (Å)	Crysralline size (nm)
Bi_2_WO_6_ nanostructures	28.06°	131	1.634	3.176	5
	2	200	1.337	2.743	6
	46.90°	201	1.391	1.935	6
Bi_2_WO_6_/MWCNTs	28.47°	131	0.5033	3.13	28
Nanocomposite at molar ratio 1:2.5	32.97°	200	0.4603	2.713	31
	47.25°	201	0.5038	1.922	30

**Fig 2:**
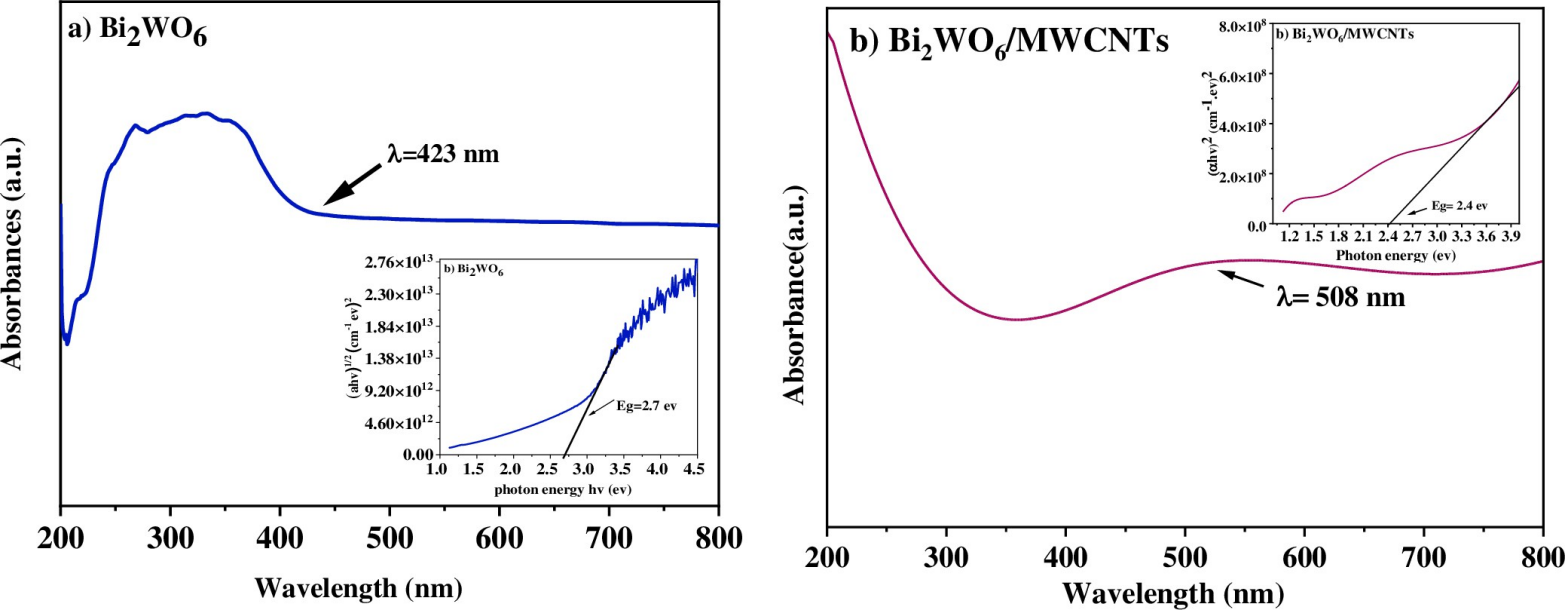
Optical absorbance spectra of a) Bi_2_WO_6_ and b) Bi_2_WO_6_/MWCNTs and inset figures plot of the variation of (αhυ)^1/2^ vs. photon energy for pure and nanocomposite utilizing the hydrothermal technique.

From Table 1, the average crystallite size of Bi_2_WO_6_ nanostructures is about 6 nm and the standard deviation is about 0.47, while in the state of the nanocomposite, the average crystallite size is about 29.67 nm and the standard deviation is about 1.25. The ultraviolet-visible absorption spectra of pure Bi_2_WO and Bi_2_WO_6_/MWCNTs, nanocomposite were illustrated in Fig 2(A) and 2(B). Fig 2(A) of Bi_2_WO_6_ nanostructure showed a broad absorption band extended from 250 nm in the ultraviolet into the visible region up to 450 nm indicating that pure Bi_2_WO_6_ can absorb a significant portion of visible right. Besides, the redshift at 423 nm highlights its absorption capability in the visible range and it is found that the absorption band performs as a cridp photo-absorption edge in the visible region.

The optical absorbance of Bi_2_WO_6_ demonstrated that the absorption at the band gap results from the nanostructure transition (transition from valance band (VB) to the conduction band (CB)) rather than transitions from impurity levels, to determine the absorption edge energy, which is Eg = 2.7 eV inset image. Furthermore, band gap energy in inset Fig 2(A), illustrates how to compute Eg for a direct transition, the Tauc plot of (αhυ)^2^ versus photon energy (hυ) was utilized, with n = 1/2. The Bi_2_WO_6_/MWCNTs nanocomposite has an impact on the optical absorption properties, as shown in Fig 2(B). Interestingly, the Bi_2_WO_6_/MWCNTs nanocomposite exhibits a strong optical absorption peak at a wavelength of 508 nm. By using Tauc’s equation [31], the Absorption coefficient determines the nanocomposite band gap: (α.hυ)^1\n^ = B(hυ-E_g_) where β denotes a constant contingent upon the structure of the specimen, h represents Planck’s constant, E_g_ signifies the band gap energy, and n serves as an empirical index. To determine the band gap, a plot was constructed using the function (αhv)^1/n^ against hv. As illustrated in Fig 2(B), the reduction of energy band gap to 2.4 eV after embedded MWCNTs. Besides, Table 2 reveals the band gap values of Bi_2_WO_6_, and Bi_2_WO_6_/MWCNTs, nanocomposite with increasing MWCNTs at a specific molar ratio of Bi_2_WO_6_: MWCNTs 1:2.5. The addition of MWCNTs could result in more energy levels from the conduction band to the valence band, which would lower the nanocomposite’s band gap [32]. These findings indicate that either the interaction between Bi_2_WO_6_ and MWCNTs or vacancies on the MWCNTs may be the reason for the diminishing band gap of the nanocomposite [33]. Furthermore, the UV-Vis spectra analysis of the Bi_2_WO_6_/MWCNTs nanocomposite showed, that the absorption maximum (λ_max_) red-shifted to a higher wavelength, thereby reducing the energy disparity and a recommendation to increase the particle size. In addition, this narrower 2.4 eV band gap allows the nanocomposite to absorb a wider range of visible light photons more efficiently, as evident from the UV-Vis spectra, which enhanced the photocatalysitc activity of nanocomposite. Free radicals O^2-^ and OH^-^ formed in the reactions can react with organic substances inside the cell membrane to produce the cell toxins, leading to the death of the cell.

**Table 2:** The Optical band gaps of Bi_2_WO_6_, and nanocomposite Bi_2_WO_6_/MWCNTs.

Materials	Wavelength (nm)	The energy band gap (eV)
Pure Bi_2_WO_6_ before adding MWCNTs	448	2.7
Bi_2_WO_4_/MWCNTs nanocomposite	508	2.4

On the other hand, the energy band gap has decreased as a result of the quantum confinement effect of the Bi_2_WO_6_/MWCNTs nanocomposite brought on by the increase in particle size. In materials with a low electron density, the only thing that can excite electrons over the band gap is the energy difference between the conduction and valance bands when the size of the nanoparticles is high. In conclusion, the addition of MWCNTs effectively improved the forbidden bandwidth and visible light response of the Bi_2_WO_6_ [34, 35].

**Fig 3:**
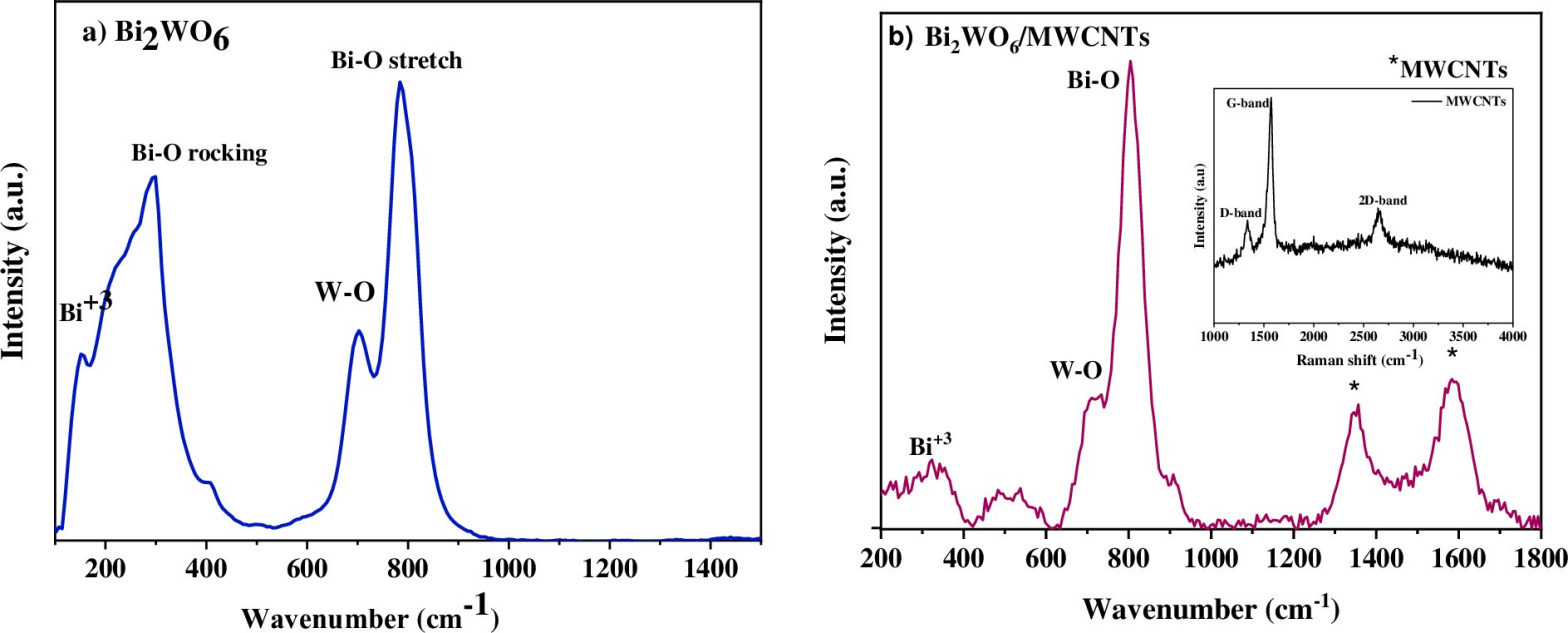
Raman spectra of a) pure Bi_2_WO_6_ and b) Bi_2_WO_6_/MWCNTs nanocomposite at a molar ratio set as 1:2.5 Bi_2_WO_6_: MWCNTs by using hydrothermal method.

Fig 3(A) and 3(B), displays the Raman spectrum of Bi_2_WO_6_ and Bi_2_WO_6_\MWCNTs at a specific molar ratio of 1:2.5 Bi_2_WO_6_: MWCNTs, respectively between the 200–1400 range cm^-1^. As seen in Fig 3(A), pure Bi_2_WO_6_ Raman peaks were related to the orthogonal Bi_2_WO_6_ [36]. The typical bands seen within a spectrum (700–800) cm^-1^ are related to the structure of W-O and Bi-O stretches. Additionally, a splattered pattern can be seen at a peak of 703 cm^-1^, which is a result of size reduction distorting the W-O contact, and a peak at 787 cm^-1^, which is associated with Bi-O, stretches [37]. The 295 cm^-1^ band may be due to translational modes resulting from the addition of CTAB, which include the simultaneous motion of WO_4_ and Bi^+3^ [38]. Similarly, the WO_6_ octahedron and the BiO_6_ polyhedron’s rocking or bending modes are primarily responsible for the vibrations in the range of (100–450) cm^-1^ for Bi_2_WO_6_. The inclusion of CTAB resulted in the splitting of the Bi-O peak at 297 cm^-1^ into more pronounced bands. It is also noticed that by introducing CTAB into Bi_2_WO_6_, the nanostructure becomes less ordered and its thickness is reduced. Consequently, it has been proposed [39, 40] that the addition of CTAB results in structural alteration that produces the WO_6_ octahedron. The recorded Raman spectra and the findings of the XRD study are in agreement [41, 42]. The Raman spectra of synthesized Bi_2_WO_6_/MWCNTs nanocomposite were presented in Fig 3(B). In the Raman spectra of Bi_2_WO_6_/MWCNTs nanocomposite, the characteristic peak was observed at 710 cm^-1^, corresponding to the symmetric mode of terminal O-W-O stretching mode within the Bi_2_WO_6_ structure. Moreover, peaks at 804 cm^-1^ were identified, showing the antisymmetric bridging mode of the tungstate chain Bi-O. Furthermore, translational modes involving the simultaneous motion of Bi^3+^ were observed at a peak of 317 cm^-1^ as depicted in Fig 3(B) [43]. In Bi_2_WO_6_/MWCNTs nanocomposites, the Raman spectra revealed the presence of the D-band, demonstrating disordered or sp^3^-hybridized carbons within the MWCNTs wall, at approximately 1356 cm^-1^ for Bi_2_WO_6_/MWCNTs. Besides, the G-band, corresponding to vibrations of sp^2^-bonded carbon atoms in graphene-like structures, such as the MWCNTs, appeared at 1588 cm^-1^ for Bi_2_WO_6_/MWCNTs nanocomposites [44]. In addition, the Raman spectrum reveals the changes in peak intensity of pure Bi_2_WO_6_ and nanocomposite after the addition MWCNTs, as shown in the Figure we find the intensity of nanocomposite decreased from I = 19128.3 at peak (787 cm^-1^) to I = 362.7 (804 cm^-1^) related to changes in the concentration of MWCNTs interaction and the presence of D-band and G-band after addition MWCNTs as compared with pure Bi_2_WO_6_.

The Raman spectroscopy results confirm that multi-walled carbon nanotubes (MWCNTs) have formed nanocomposite with Bi_2_WO_6_ in the respective sample via the hydrothermal route [45]. The D band and G band of F-MWCNTs were about 1332 cm^-1^ and 1571 cm^-1^, respectively as shown in Table 3.

**Table 3:** Raman bands assigned to Bi_2_WO_6_ and Bi_2_WO_6_\MWCNTs nanocomposite synthesized hydrothermally.

Material	Raman bands (cm^-1^)	Assignation
**Bi_2_WO_6_ before adding MWCNTs**	787	stretching of Bi-O bonds
	703	Asymmetric stretching of WO_6_
	295	Bending of Bi-O bonds
**Bi_2_WO_6_/MWCNTs nanocomposite**	804	stretching of Bi-O bonds
	710	Asymmetric stretching of WO_6_
	317	Bending of Bi-O bonds
	1356	*D-band of MWCNTs
	1588	*G- band of MWCNTs

Where *D and *G represent the Raman peaks of MWCNTs before addition.

**Fig 4:**
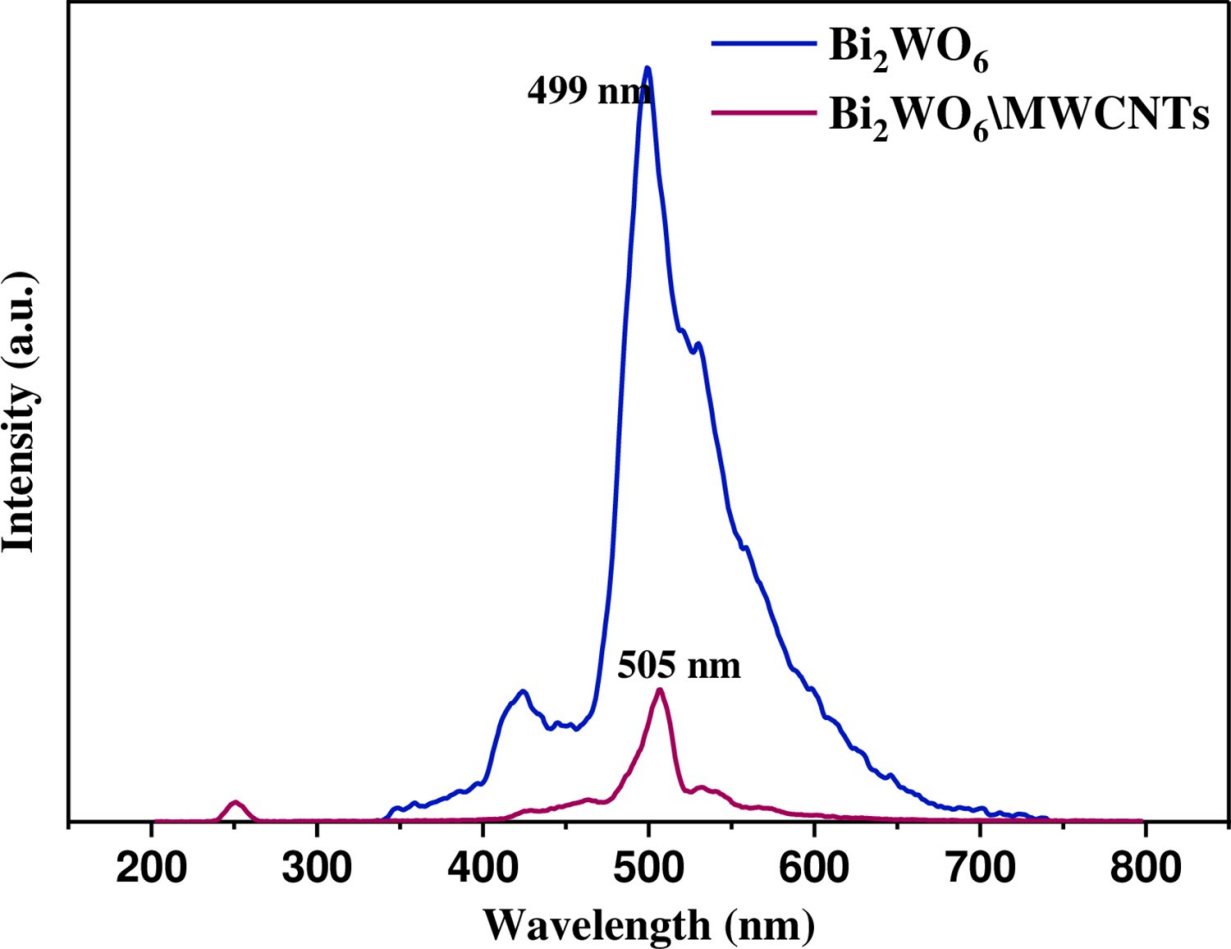
PL spectrum of pure Bi_2_WO_6_ (blue line) and Bi_2_WO_6_/MWCNTs nanocomposite (pink line) at a molar ratio of 1:2.5 Bi_2_WO_6_: MWCNTs by using hydrothermal method.

The analysis of photoluminescence (PL) spectra is a crucial aspect of evaluating the optical characteristics of materials for photocatalytic applications that are pure Bi_2_WO_6_ and Bi_2_WO_6_/MWCNTs nanocomposite. PL analysis offers valuable insights into charge carrier recombination and photocatalytic efficiency in visible regions [46]. In Fig 4, the PL spectrum of samples had two relatively obvious peaks in response to a 250 nm excitation wavelength. As shown in Fig 4, the high intensity of emission PL (I = 761.7 at λ = 499nm) indicates a fast rate of charge recombination of pure Bi_2_WO_6_ semiconductor photocatalyst (blue line). In the case of Bi_2_WO_6_ \MWCNTs nanocomposite at a molar ratio of set as 1:2.5 Bi_2_WO_6_: MWCNTs, The PL exhibits weak intensity (I = 128.6 at λ = 505nm), as displayed in Fig 4. This is indicative of electron-hole pair recombination that occurs at a low rate (pink line). The reason that the photogenerated (e-h) pairings have a lower recombination rate was linked to the nanocomposite’s lower PL intensity. In other words, the resulting nanocomposite was observed to exhibit a significantly weaker PL emission peak than pure Bi_2_WO_6_ which was in accord with the observed red shift in the absorption band edge of UV.Vis spectra. Besides, the addition of MWCNTs resulted in a decreased energy gap of Bi_2_WO_6_ and led to redshifts of absorption band edges, thereby enhancing the visible light response. As a result, the MWCNTs addition was deduced to have modified the Bi_2_WO_6_ morphology as revealed by FESEM and TEM and promoted surface photogenerated (e-h) separated, thus efficiently inhabited photo-carrier recombination as confirmed by PL [47–52].

**Fig 5:**
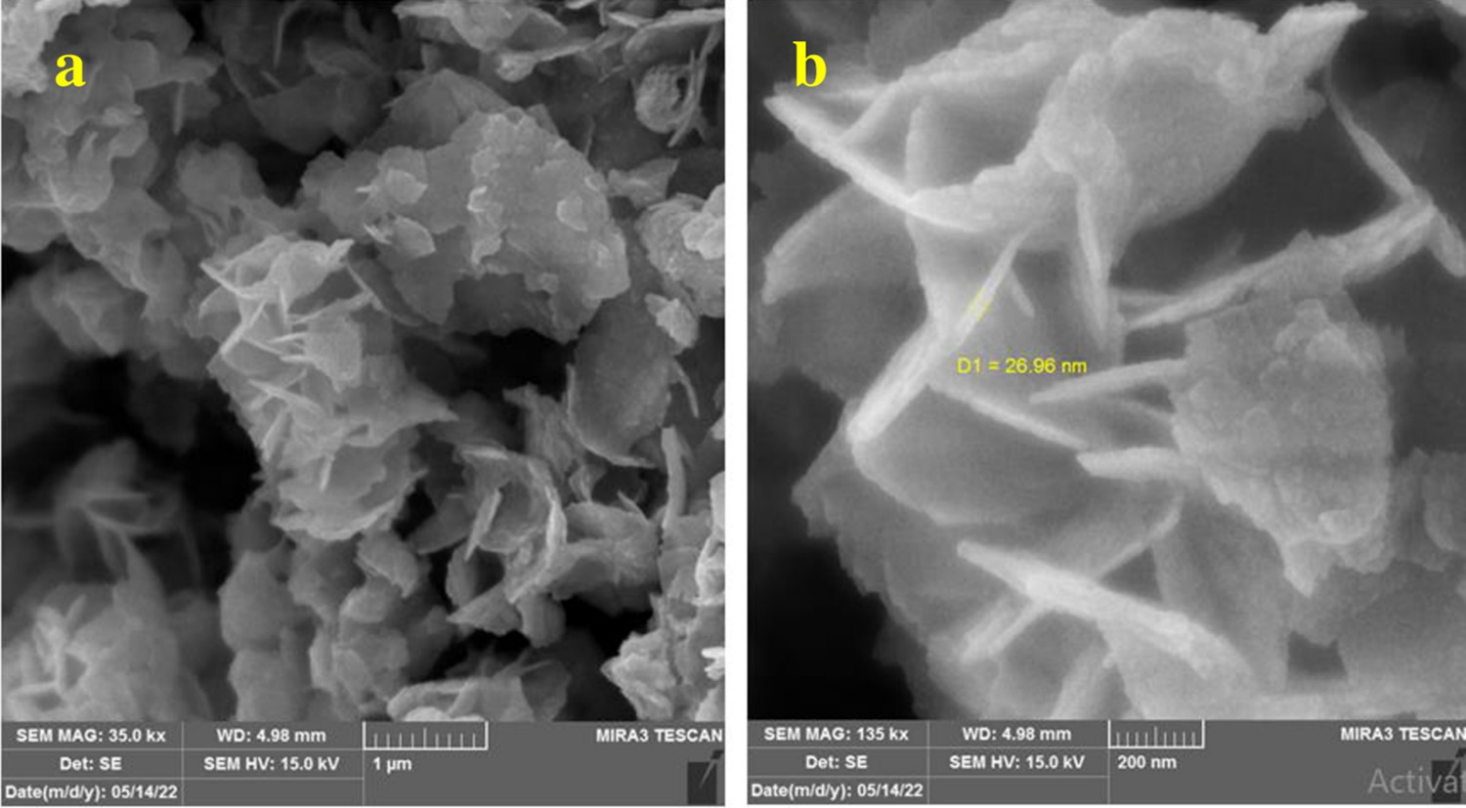
FESEM images of pure Bi_2_WO_6_ with different magnifications a) at scale bar = 2μm, b) scale bar = 200 nm.

**Fig 6:**
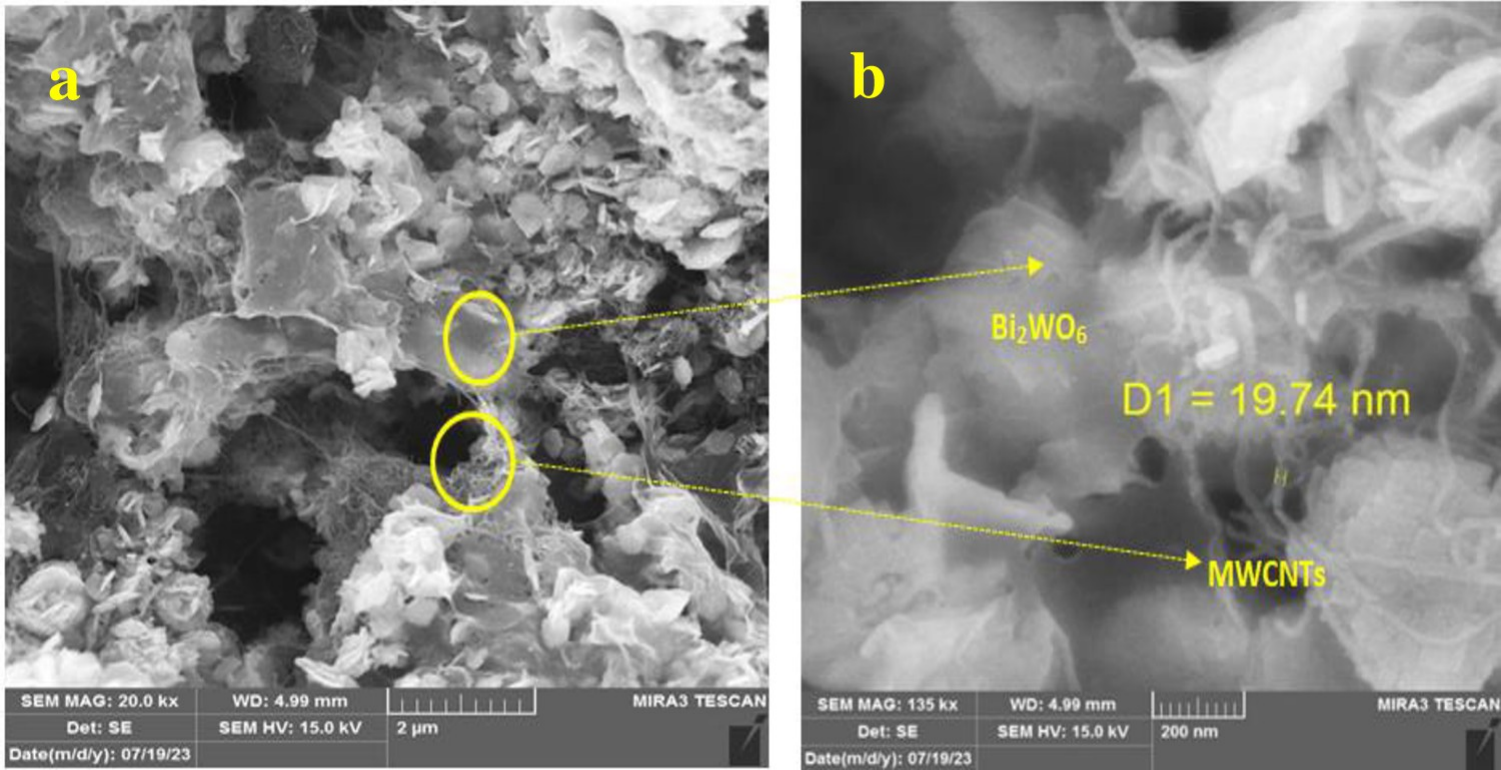
FESEM images of pure Bi_2_WO_6_\MWCNTs nanocomposite with different magnifications a) at scale bar = 2μm, b) scale bar = 200 nm.

Fig 5(A) and 5(B) presents the FESEM morphology of Bi_2_WO_6_ and Bi_2_WO_6_/MWCNTs nanocomposite at a molar ratio of 1:2.5 Bi_2_WO_6_: MWCNTs, respectively. In Fig 5(A) and 5(B), the images reveal the presence of uniform spherical aggregates composed of clusters and crystal structures resembling layered nanoparticles. Besides, the results of FESEM images indicate 2D Bi_2_WO_6_ nanoflakes morphology which agglomerated with each other. The diameter of each particle as seen in magnified 200 nm is around 1–2μm and the resulting particles of pure Bi_2_WO_6_ are related to Gibbs-Thomson theory represented by a significant nucleation and decrease in the total energy of the system. This approach is responsible for high surface area with large size at hydrothermal method. Furthermore, in FESEM of Bi_2_WO_6_/MWCNTs, the MWCNTs dispersed in the Bi_2_WO_6_ nanostructure surface verify that there is a strong contact and binding between MWCNTs and Bi_2_WO_6_ nanostructure As may be seen in Fig 6(A) and 6(B). It’s found that increasing the MWCNTs at a specific molar ratio 1:2.5 of Bi_2_WO_6_: MWCNTs resulted in embedded inside and on the surface of Bi_2_WO_6_ as shown in FESEM images. MWCNTs serve as substrates for the development of nanoparticles. It is worth noting, that the addition of the optimum quantity of MWCNTs prevents Bi_2_WO_6_ nanoparticles from aggregating and increasing the surface area [47]. Moreover, the reduction of high-energy exposed surfaces or attractive forces between particles may be used to explain the agglomeration. This is because particle size has an impact on surface energy between particles and between particles and solvents, which may result in the development of agglomerates and irregular distribution of particles as previously indicated [53]. These results reveal a reduction of particle size of nanocomposite compared to pure Bi_2_WO_6_ because of the tendency to agglomeration instead of dispersing homogenously in the nanocomposite.

**Fig 7:**
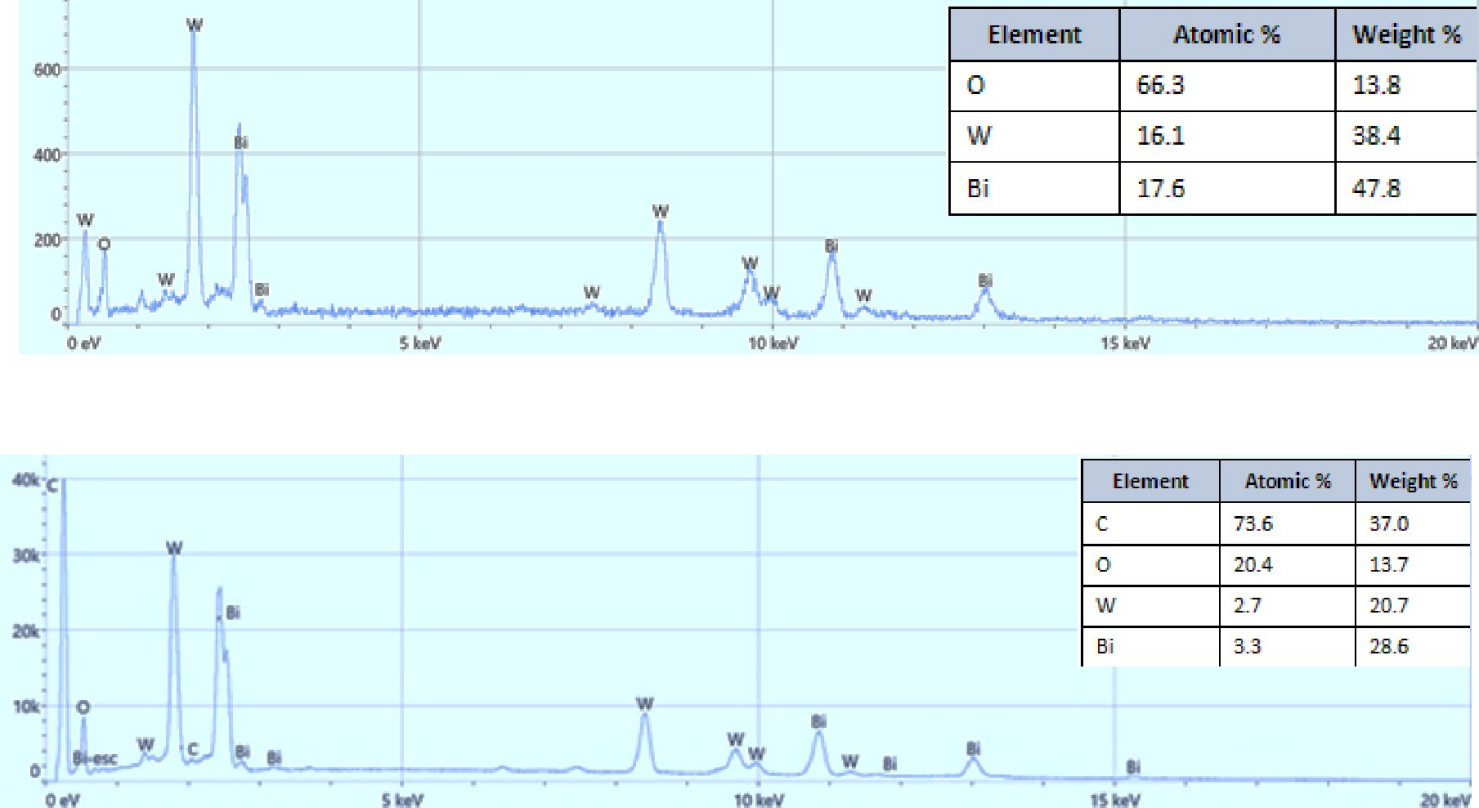
EDX spectrum of pure Bi_2_WO_6_ (Upper panel), and Bi_2_WO_6_/MWCNTs nanocomposites (Lower panel).

**Fig 8:**
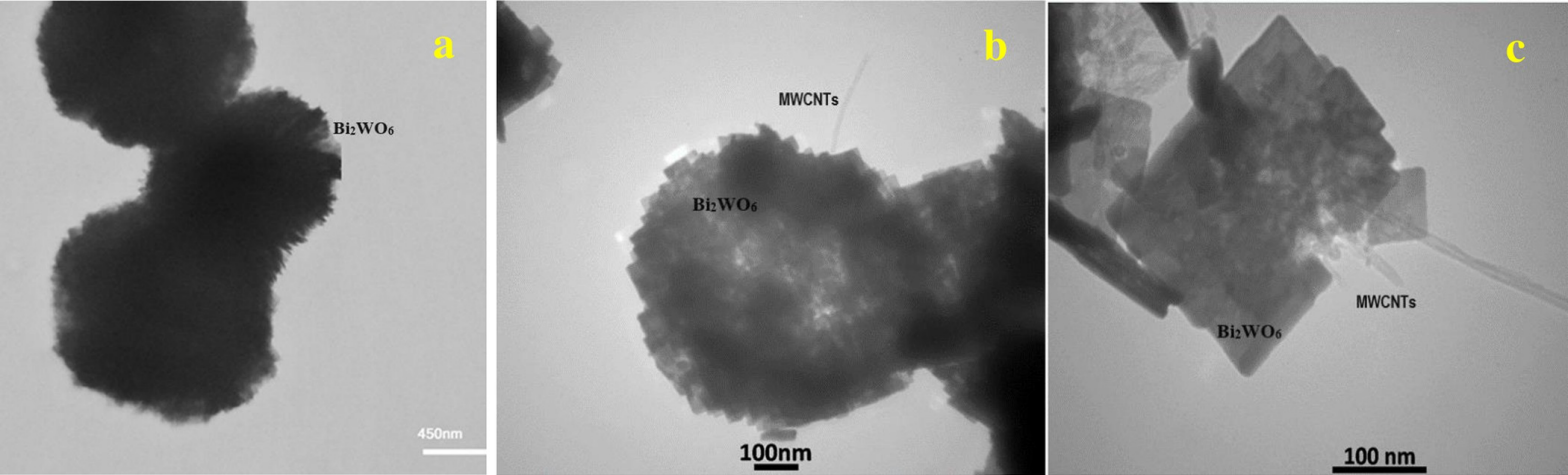
TEM images of the a) pure Bi_2_WO_6_ and b, c) Bi_2_WO_6_\MWCNTs nanocomposite at different magnified 100 nm.

The EDS technique was employed to estimate the chemical composition of the resulting flower-like structure of pure Bi_2_WO_6_ as seen in Fig 7. The outcomes reveal that the product made up of only the Bi, W, and O elements signals occurred in the flower-like structure of Bi_2_WO_6_. Fig 7 (Upper panel) shows further characterization of nanocomposite at a specific molar ratio of 1:2.5 Bi_2_WO_6_: MWCNTs, the results from the EDS pattern analysis verified that the elements Bi, O, W, and C were present within the sample Fig 8 (Lowe panel). Besides, the results indicate a strong peak of C in the nanocomposite about 73.6 as compared with Bi which is about 3.3 due to the high content of MWCNTs in the nanocomposite at a specific molar ratio of 1:2.5 Bi_2_WO_6_: MWCNTs.

The morphological and structural properties of the pure Bi_2_WO_6_, and Bi_2_WO_6_/MWCNTs, nanocomposite at a specific molar ratio of 1:2.5 Bi_2_WO_6_: MWCNTs were examined at various magnified using the transmission electron microscopy (TEM) images, as shown Fig 8(A)–8(C), respectively. In Fig 8(A), the TEM images reveal the whole and individual microspheres of the flower-like structure pure Bi_2_WO_6_, with an average diameter of around 1–2 μm. The results show that pure Bi_2_WO_6_ can be manufactured effectively, which is in agreement with the XRD analysis. In the state of Bi_2_WO_6_/MWCNTs nanocomposite at a specific molar ratio of 1:2.5 Bi_2_WO_6_ the TEM images displayed more detailed information on the microstructure and morphology as seen in Fig 8(B) and 8(C). nanocomposite at different magnified 100 nm.

Moreover, the TEM images of Bi_2_WO_6_/MWCNTs nanocomposite showed MWCNTs were coiled around the Bi_2_WO_6_ surface and some of them embedded in Bi_2_WO_6_, which is in contrast to the comparatively smooth surface of free MWCNTs. The results are consistent with the FESEM images, which show that MWCNTs have a small, discernible impact on the morphology of the Bi2WO6 structure. Increasing the number of MWCNTs to 2.5% easily induces an agglomeration of MWCNTs in the Bi2WO6/MWCNTs matrix as shown in TEM. It is worth noting, that MWCNTs demonstrated effective electron transport and this feature had a great effect on the transmission of photogenerated electrons in Bi_2_WO_6_ as improved by PL measurement of Bi_2_WO_6_/MWCNTs nanocomposite. Mainly, the surface of the nanocomposite shows a variety of contact surfaces between Bi_2_WO_6_ and MWCNTs. Through hydrothermal methods which made MWCNTs easy to embed in the gaps of nanoparticles. Moreover, a successful and widespread method to avoid nanotube aggregation, functionalizing MWCNTs helps with greater dispersion and stabilization. Similar experimental results also appeared in previous studies [54–59].

### 3.2 Bi_2_WO_6_/MWCNTs nanocomposite increases autophagy in BMDMs

**Fig 9:**
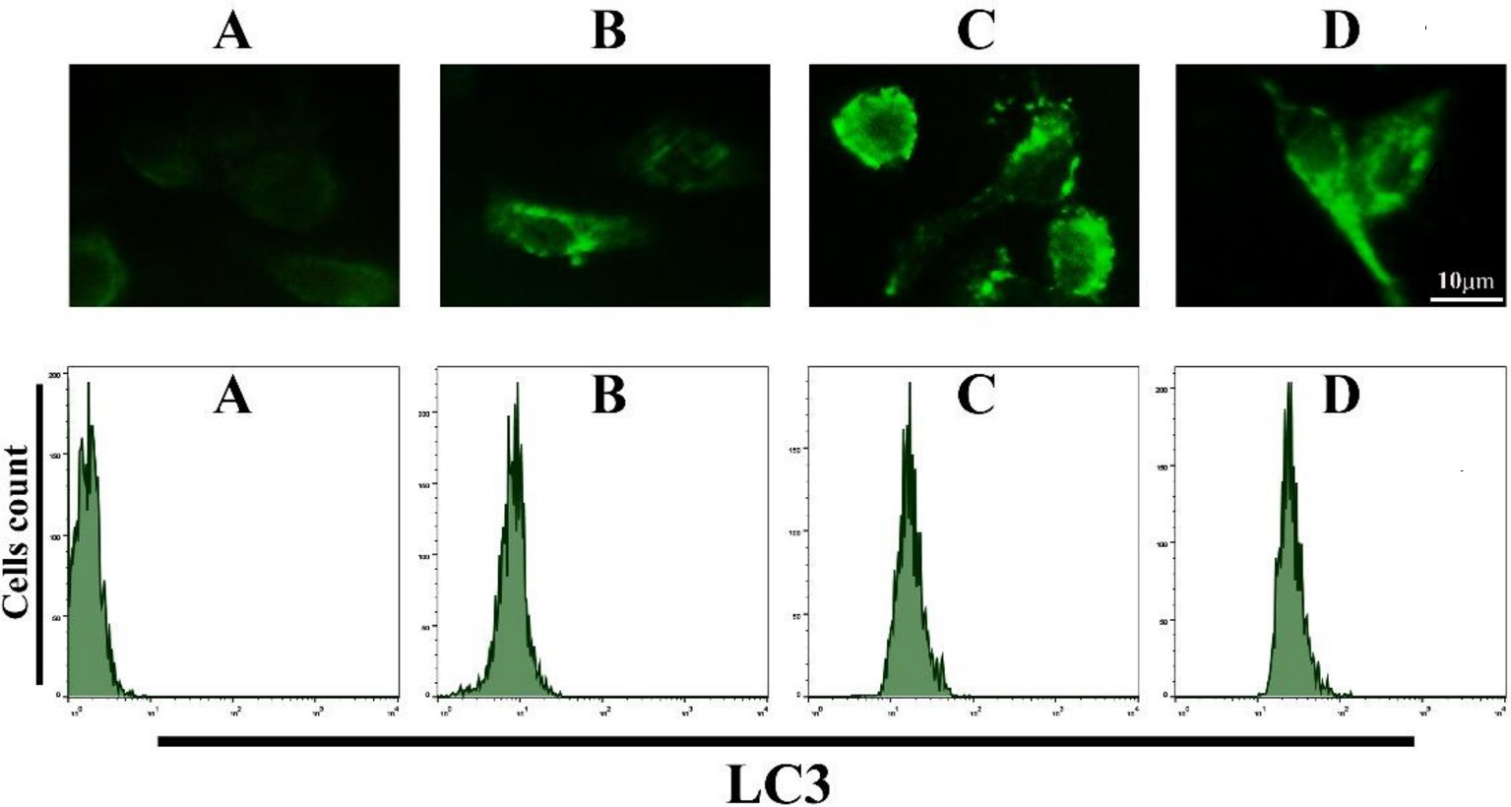
Bi_2_WO_6_/MWCNTs augmented autophagy in BMDMs. A Control untreated BMDMs. B, LPS+ATP treated BMDMs. C, LPS+ATP treated BMDMs in the presence of Bi_2_WO_6_. D, LPS+ATP treated BMDMs in the presence of Bi_2_WO_6_/MWCNTs.

We hypothesized that Bi_2_WO_6_/MWCNTs will augment autophagy. Thus, Bi_2_WO_6_/MWCNTs’ impact on the autophagy mechanism was tested. In the current study, BMDMs were given LPS+ATP treatment both with and without Bi_2_WO_6_/MWCNTs. The ability of Bi_2_WO_6_/MWCNTs to boost autophagy, as shown by the LC3 autophagosomes’ presence. as indicated in Fig 9 (Upper panel). The induction of autophagy by Bi_2_WO_6_/MWCNTs was investigated by calculating the LC3 production protein using a flow cytometric assay as shown in Fig 9 (Lower panel). The present study’s findings demonstrated that Bi_2_WO_6_/MWCNTs augmented the degree of autophagy. In recent years, researchers have shown that a range of nanomaterials may trigger autophagy [11]. Furthermore, earlier research [60] indicated that MWCNTs are evolving into a class of inducers of autophagy. It was shown that the production of ROS can send out a signal that may be used to upregulate SIRT1, One of the class III histone deacetylases reliant on NAD [61]. Furthermore, AgNP-induced disruption of lysosomes, which may result in a rise in internal acidity or a loss of membrane integrity, is linked to a modified autophagosome-lysosome fusion mechanism [62], which seriously impairs the function of the autophagy machinery.

### 3.3 Bi_2_WO_6_/MWCNTs NLRP3 inflammasome and ASC activation

**Fig 10:**
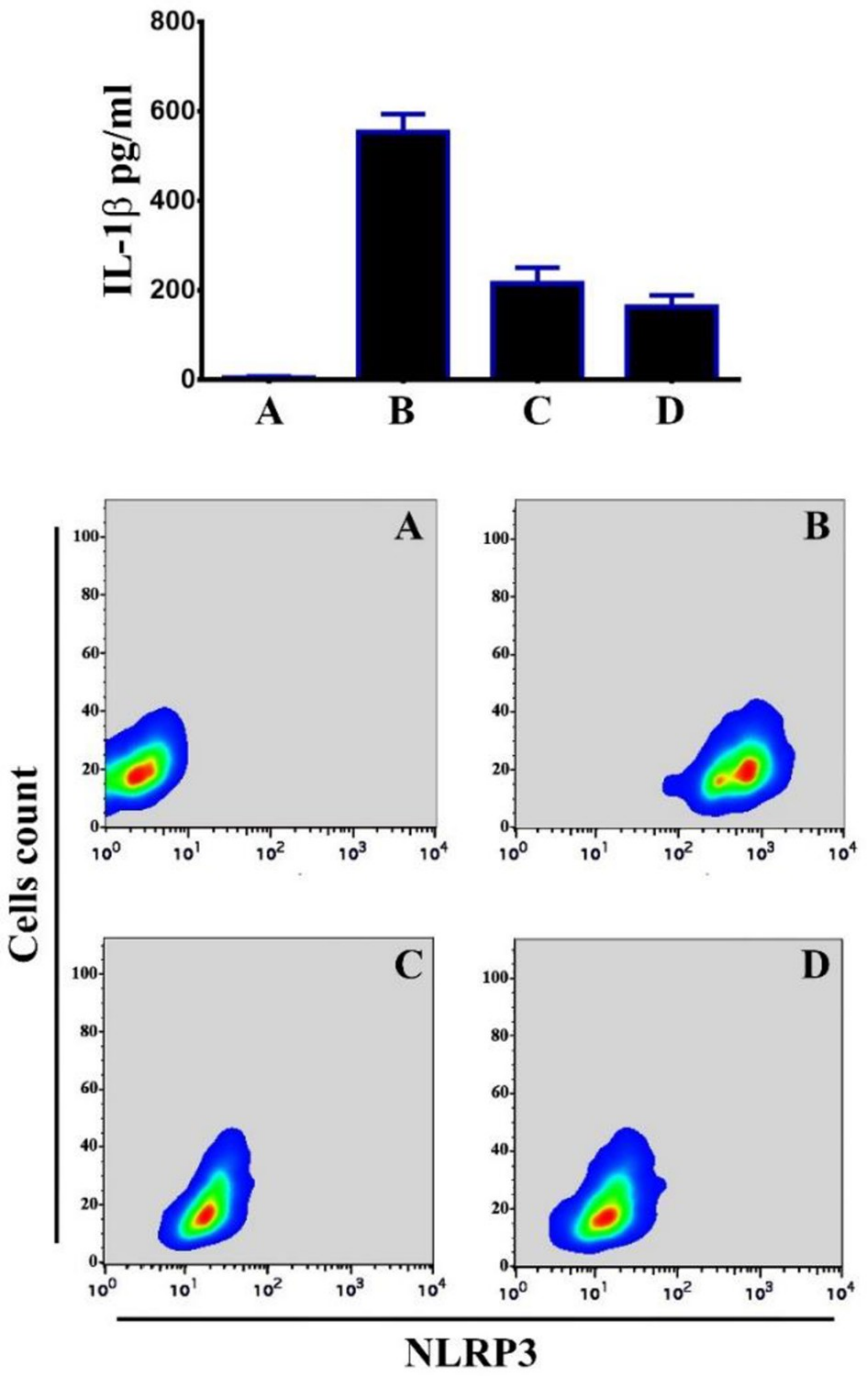
Bi_2_WO_6_/MWCNTs reduce NLRP3 inflammasome activation. A Control untreated BMDMs. B, LPS+ATP treated BMDMs. C, LPS+ATP treated BMDMs in the presence of Bi_2_WO_6_. D, LPS+ATP treated BMDMs in the presence of Bi_2_WO_6_/MWCNTs.

**Fig 11:**
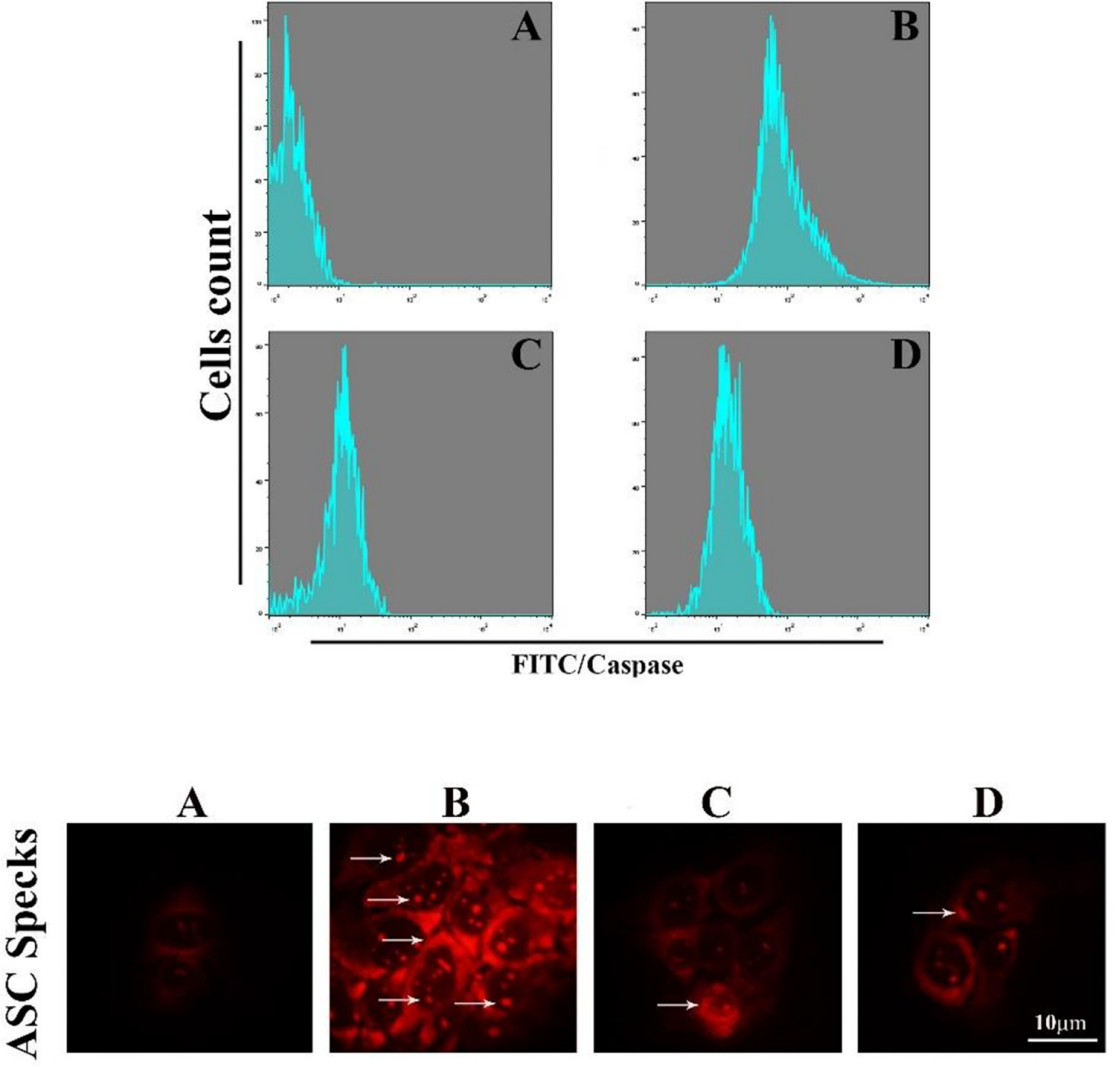
Bi_2_WO_6_/MWCNTs reduce caspase activation and ASC oligomerization. A Control untreated BMDMs. B, LPS+ATP treated BMDMs. C, LPS+ATP treated BMDMs in the presence of Bi_2_WO_6_. D, LPS+ATP treated BMDMs in the presence of Bi_2_WO_6_/MWCNTs.

We hypothesized that Bi_2_WO_6_/MWCNTs nanocomposite augmented autophagy could inhibit the activation of inflammatory caspases. When BMDM cells were pretreated with Bi_2_WO_6_/MWCNTs nanocomposite after applying LPS and ATP to the nanocomposite, the results demonstrated reductions in IL-1 levels as indicated in Fig 10 (Upper panel), and reduction in the level of NLRP3 as in Fig 10 (Lower panel). Inflammasome activation is dependent on two signals. First, via NF-B induction, which causes pro-IL-1 and pro-caspase-1 to be produced. Second, is necessary for the inflammasome complex to properly assemble, which triggers the recruitment of pro-caspase-1. In the current study, BMDMs cells were exposed to LPS + ATP in the presence and absence of Bi_2_WO_6_/MWCNTs nanocomposite to investigate the effect of Bi_2_WO_6_/MWCNTs nanocomposite on Capase-1 activation and ASC oligomerization. The findings demonstrated that Bi_2_WO_6_/MWCNTs nanocomposite can reduce Caspae-1 activity as in Fig 11 (Upper panel). Remarkably, pre-treating BMDMs cells with Bi_2_WO_6_/MWCNTs nanocomposite, and then exposure to LPS+ATP suggests that Bi_2_WO_6_/MWCNTs nanocomposite may inhibit the release of IL-1 by interfering with signal. In addition, we examined the inhibitory effect of Bi_2_WO_6_/MWCNTs nanocomposite on the adaptor protein ASC after receiving LPS+ATP therapy. We used the immunofluorescence assay to assess ASC specks with NLRP3 inflammasome complexes to investigate this activity. ASC specks were more recognized in cells treated with LPS+ATP. Nevertheless, there were noticeably fewer ASC specks in the cells treated with Bi_2_WO_6_/MWCNTs nanocomposite as shown in Fig 11 (Lower panel). Collectively, the findings showed that Bi_2_WO_6_/MWCNTs nanocomposite block ASC oligomerization, which prevents the inflammasomes from activating.

### 3.4 Bi_2_WO_6_/MWCNTs enhance autophagy’s targeting of NLRP3

**Fig 12:**
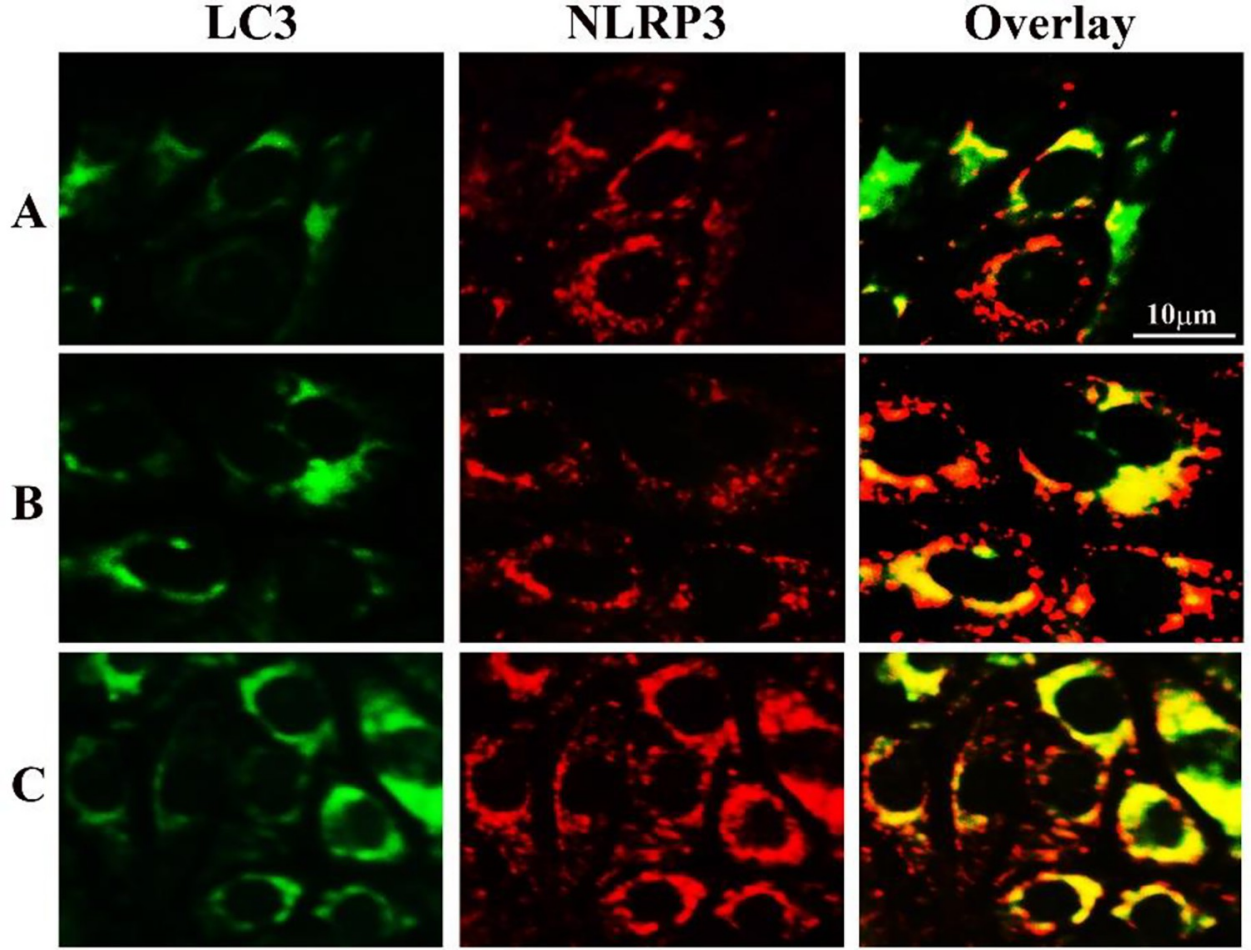
Bi_2_WO_6_/MWCNTs nanocomposite increases targeting of NLRP3 by autophagy. A, LPS+ATP treated BMDMs. B, LPS+ATP treated BMDMs in the presence of Bi_2_WO_6_. C, LPS+ATP treated BMDMs in the presence of Bi_2_WO_6_/MWCNTs.

In the current study, Staining of BMDM cells treated with LPS+ATP was done using anti-NLRP3 and anti-LC3 antibodies. The results were visualized under confocal microscopy to investigate the intracellular localization of NLRP3 and how autophagy regulates its pattern of secretion. The results confirmed that Fig 12 illustrates the significant co-localization that occurs between LC3 and NLRP3 when Bi_2_WO_6_/MWCNTs are present. These results show that the Bi_2_WO_6_/MWCNTs stimulate the degradation of NLRP3 inflammasome and IL-1~β, in addition to increasing autophagy. Consequently, autophagy plays a crucial role in regulating the NLRP3 inflammasome and IL-1β release. A study by Yang M. et al [63], demonstrated that the purified CNTs and CNOs that have functionalized by benzoic acid have significantly reduced immune cytokine IL-1 β release, and a marked decline in the number of neutrophils and monocytes recruited after injection of prepared nanoparticles in mice (*In-vivo* model study).

### 3.5 Inhibited autophagy reduces the role of Bi_2_WO_6_/MWCNTs nanocomposite in inflammasome activation

**Fig 13:**
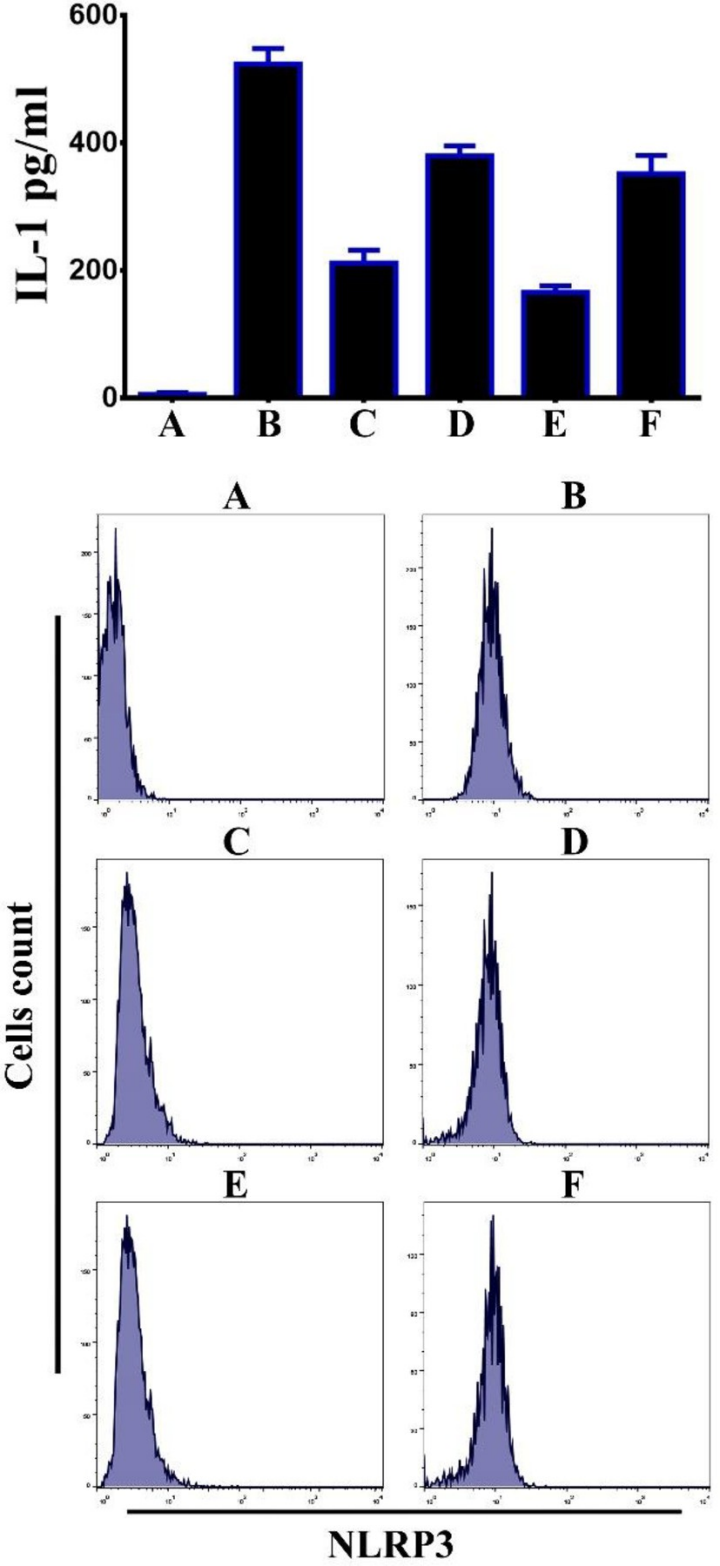
Inhibition of autophagy increased inflammasome activation. A Control untreated BMDMs. B, LPS+ATP treated BMDMs. C, LPS+ATP treated BMDMs in the presence of Bi_2_WO_6_. D, LPS+ATP treated BMDMs in the presence of Bi_2_WO_6_ + 3MA. E, LPS+ATP treated BMDMs in the presence of Bi_2_WO_6_/MWCNTs. F, LPS+ATP treated BMDMs in the presence of Bi_2_WO_6_/MWCNTs+3-MA.

In the present investigation, we additionally examined the impacts of autophagy inhibition on the activation of the inflammasome in BMDMs cells that were treated with Bi_2_WO_6_/MWCNTs nanocomposite following LPS+ATP treatment in the existence or lack of the autophagy inhibitor 3-methyladenine (3-MA). The activated NLRP3-inflammasome and the amount of IL-1β secreted in BMDM cells treated with LPS+ATP and Bi_2_WO_6_/MWCNTs nanocomposite combined were both elevated, according to the findings. As illustrated in Fig 13, the results showed that there was a significant increase in the levels of IL-1 β, and NLRP3. These results show that, after Bi_2_WO_6_/MWCNTs nanocomposite, and Treatment of LPS+ATP in the presence of 3-MA, increased inflammasome activation results from the blocked autophagy.

Nanoparticles are substances that have unique physiological properties with vast applications in various fields [64]. They are becoming more significant every day because of their profound effect on human health [65]. Nanoparticles are widely used against pathogens [66, 67], to improve drug delivery [68] and are considered a promising tool for various treatment purposes [69]. These nanoparticles can be synthesized from various plants, metals, microorganisms, and organic and inorganic compounds [70, 71].

## 4. Conclusions

The pure Bi_2_WO_6_ and Bi_2_WO_6_\MWCNTs nanocomposite at a specific molar ratio of 1:2.5 Bi_2_WO_6_: MWCNTs were successfully synthesized via the hydrothermal method. Adding MWCNTs to Bi_2_WO_6_ led to some key effects improved crystallinity, reduced band gap energy, lower photogenerated charge carriers’ combination rates, and reduced nanoparticle aggregation as confirmed by XRD, and UV. visble, Raman, PL, FSEM\EDS and TEM. The results reveal a strong interfacial contact and binding between the MWCNTs and Bi_2_WO_6_ nanostructures within the nanocomposite. Overall, the synergistic combination of MWCNTs with Bi_2_WO_6_ creates functional nanomaterials. Additionally, Bi_2_WO_6_/MWCNTs nanocomposite increased autophagy and decreased NLRP3 inflammasome activation and IL-1β levels in BMDMs. This suggested that Bi_2_WO_6_/MWCNTs augment autophagy which leads to targeting IL-1 in two ways. The first one controls the activation of caspase-1, IL-1, and ASC, and the other involves lysosomal degradation of NLRP3. Taken together, these results increase the opportunity that Bi_2_WO_6_/MWCNTs nanocomposite may be a powerful treatment for controlling inflammation by enhancing autophagy.
